# Plant-Derived Antimalarial Agents: New Leads and Efficient Phytomedicines. Part II. Non-Alkaloidal Natural Products

**DOI:** 10.3390/molecules14083037

**Published:** 2009-08-13

**Authors:** Ronan Batista, Ademir de Jesus Silva Júnior, Alaíde Braga de Oliveira

**Affiliations:** 1 Departamento de Estudos Básicos e Instrumentais, Universidade Estadual do Sudoeste da Bahia – UESB, BR 415, Km 03, s/nº, 45.700-000 Itapetinga, BA, Brazil; E-mail: ademirjr18@yahoo.com.br (A.d.J.S.J.); 2 Departamento de Produtos Farmacêuticos, Faculdade de Farmácia, Universidade Federal de Minas Gerais, Av. Antônio Carlos, 6627, 31270-901 Belo Horizonte, MG, Brazil; E-mail: alaidebraga@terra.com.br (A.B.d.O.)

**Keywords:** malaria, non-alkaloidal natural products, *Plasmodium*, antiplasmodial activity

## Abstract

Malaria is still the most destructive and dangerous parasitic infection in many tropical and subtropical countries. The burden of this disease is getting worse, mainly due to the increasing resistance of *Plasmodium falciparum* against the widely available antimalarial drugs. There is an urgent need for new, more affordable and accessible antimalarial agents possessing original modes of action. Natural products have played a dominant role in the discovery of leads for the development of drugs to treat human diseases, and this fact anticipates that new antimalarial leads may certainly emerge from tropical plant sources. This present review covers most of the recently-published non-alkaloidal natural compounds from plants with antiplasmodial and antimalarial properties, belonging to the classes of terpenes, limonoids, flavonoids, chromones, xanthones, anthraquinones, miscellaneous and related compounds, besides the majority of papers describing antiplasmodial crude extracts published in the last five years not reviewed before. In addition, some perspectives and remarks on the development of new drugs and phytomedicines for malaria are succinctly discussed.

## 1. Introduction

Among all parasitic agents causing disease in humans, malaria is undoubtedly the single most destructive and dangerous infectious agent in the developing world [1,2]. This vector-borne infectious disease is a classic example of one that affects the productivity of individuals, families and the whole society, since it causes more energy loss, more debilitation, more loss of work capacity and more economic damage than any other human parasitic diseases [3]. Malaria is commonly associated with poverty, but is also a cause of poverty and a major hindrance to economic development. There were an estimated 247 million malaria cases among 3.3 billion people at risk in 2006, causing nearly a mil­lion deaths, mostly of children under 5 years of age. It is widespread in tropical and subtropical regions, including parts of the Americas, Asia and Africa. A total of 109 countries were endemic for malaria in 2008, 45 within the WHO Afri­can region [4].

**Figure 1:**
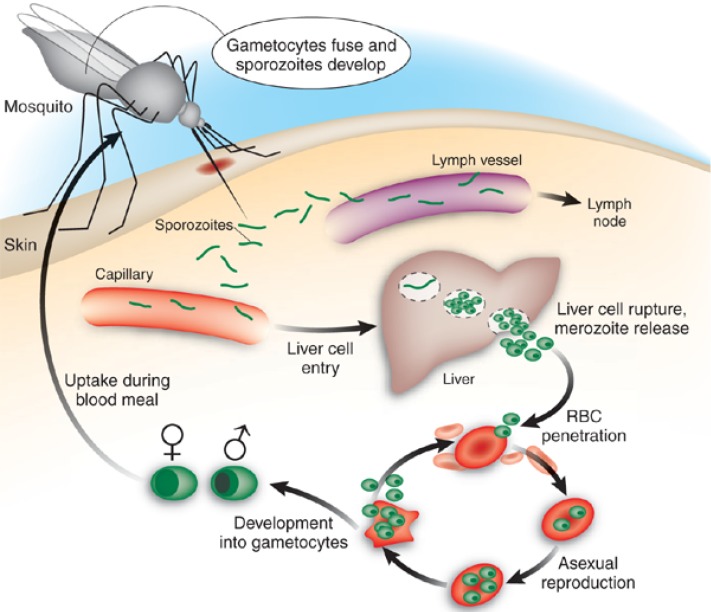
Schematic life cycle of malaria in humans. Sporozoites are injected into human dermis through the bite of infected *Anopheles* mosquito. After inoculation, sporozoites migrate to liver cells to establish the first intracellular replicative stage. Merozoites generated from this exoerythrocytic phase then invade erythrocytes (RBCs), and it is during this erythrocytic stage that severe conditions of malaria occur. The life cycle is completed when sexual stages (gametocytes) are ingested by a mosquito. Some sporozoites deposited in the skin eventually penetrate capillaries or lymph vessels. Those entering the lymph vessels will penetrate lymph vascular endothelial cells in lymph nodes to establish a lymph node form, which appears not to continue the life cycle - but may be significant in priming an immune response. Adapted and reproduced by permission from Macmillan Publishers Ltd. [7].

Although human malaria transmitted by female *Anopheles* mosquitoes has four *Plasmodium* species as its aetiological agents – *P. falciparum*, *P. vivax*, *P. ovale* and *P. malariae*, the most widespread and severe disease is caused by *P. falciparum*, which transiently infects the liver before invading red blood cells of the mammalian host (Figure 1). Clinical manifestations occur at the erythrocytic stage and can include fever, chills, prostration and anaemia, as well as delirium, metabolic acidosis, cerebral malaria and multi-organ system failure, which may be followed by coma and death [5,6,7].

Quinine (**1**), an aminoquinoline alkaloid isolated from the bark of *Cinchona* species (Rubiaceae) in 1820 by Pelletier and Caventou, is one of the oldest and most important antimalarial drugs and is still used today. For almost three centuries, this alkaloid was the sole active principle effective against *Plamodium falciparum*, and it has been considered the responsible, after the Second World War, for the development of synthetic antimalarial drugs belonging to the classes of 4- and 8-aminoquinolines, such as chloroquine (**2**, 1940) and primaquine (**3**, 1952), among others. Until recently, chloroquine (**2**) was the only drug used for the treatment of malaria [8,9].

The appearance of drug-resistance *P. falciparum* strains since 1960, in particular to chloroquine, has made the treatment of malaria increasingly problematic in virtually all malarious regions of the world [2]. Several researchers have dedicated efforts to the development of new active compounds, especially from artemisinin (**4**), as an alternative to chloroquine (**2**). Currently no single drug is effective for treating multi-drug resistant malaria, and effective combination therapy includes artemisinin derivatives such as artesunate (**5**), or mixtures with older drugs such as the atovaquone (**6**) – proguanil (**7**) combination Malarone^®^ [2,10]. Unfortunately first reports on drug resistance to artemisinin-derivatives [11] and to drug combination therapies [12] have already appeared. So, in the absence of a functional, safe and widely available malaria vaccine, efforts to develop new antimalarial drugs continue being urgently needed now.

There is a consensus among the scientific community that natural products have been playing a dominant role in the discovery of leads for the development of drugs for the treatment of human diseases [13]. Indeed, the vast majority of the existing antimalarial chemotherapeutic agents are based on natural products, and this fact anticipates that new leads may certainly emerge from the tropical plant sources, since biological chemodiversity continues to be an important source of molecular templates in the search for antimalarial drugs [14,15,16].

Few reviews focused on natural products with antiplasmodial activities have been reported in the recent literature. Saxena and co-workers published in 2003 a review article providing a critical account of crude extracts, essential oils and antiplasmodial secondary metabolites with diverse chemical structures from higher plants, collected from the period 1993-2003. A total of 127 alkaloids, 18 quassinoids, 23 sesquiterpenes, 27 triterpenoids, 21 flavonoids/xanthones, nine quinones and 25 miscellaneous compounds were highlighted in their work [8]. The review published by Frederich and collaborators in 2008 covers 31 indole alkaloids isolated from natural sources with high antiplasmodial activity (*in vitro* and *in vivo*), most of them displaying IC_50_ values under the micromolar range and with a good selectivity index [17]. Kaur and co-authors published in 2009 a review focusing on antimalarial compounds discovered during 1998-2008 from all natural sources, including crude plant and marine extracts. A total of 266 antiplasmodial natural products pertaining to the classes of alkaloids, terpenes, quassinoids, flavonoids, limonoids, chalcones, peptides, xanthones, quinones, coumarins and miscellaneous compounds, as well as 37 promising semisynthetic antimalarials, were listed in this compilation [18]. 

**Figure 2:**
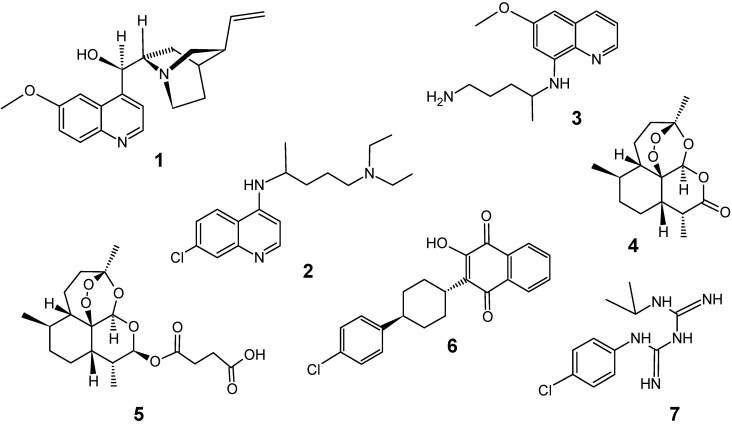
Some traditional antimalarial drugs.

Fortunately, an increasing number of articles reporting antiplasmodial natural products from plants have been published recently. Considering that a review submitted by Oliveira and co-workers in 2009 deals with plant-derived antimalarial agents pertaining only to alkaloids [19], this review is intended as a continuation of that work, covering most of all recently-published reports of non-alkaloidal natural compounds with antiplasmodial and antimalarial properties from the period 2008-2009 not reviewed yet, in addition to recently published active crude extracts.

Regarding the criteria for considering the *in vitro* antiplasmodial activity of a given extract or compound as “good”, “moderate”, “low” or “inactive”, earlier studies by Basco and co-workers [20,21] have adopted the following criteria: IC_50_ < 10 µg/mL, good activity; IC_50_ of 10-50 µg/mL, moderate activity; IC_50_ of 50-100 µg/mL, low activity; and IC_50_ > 100 µg/mL, inactive. On the other hand, Muriithi and collaborators have expressed their IC_50_ values in µM and considered as inactive compounds showing IC_50_ > 100 µM; of limited (moderate?) activity, those with IC_50_ of 1–20 µM; and of low activity those displayng IC_50_ of 20–60 µM [22]. In this paper, both criteria have been combined in order to establish the following criteria adopted for all compounds described in this review: IC_50_ < 1 µM, excellent/potent activity; IC_50_ of 1-20 µM, good activity; IC_50_ of 20-100 µM, moderate activity; IC_50_ of 100-200 µM, low activity; and IC_50_ > 200 µM, inactive. In addition, Basco and co-workers’ criteria have been used for classifying the antiplasmodial activity of crude extracts.

## 2. Recently-Published Crude Extracts with Antiplasmodial Activity

Although many crude plant extracts with *in vitro* and *in vivo* antiplasmodial activities have been reported in the recent literature, only those which were not referred to in previously published reviews [8,18,23,24] are listed in Table 1. In addition, articles with extensive results [25,26,27,28,29,30,31,32,33,34,35,36,37,38,39,40,41,42] were not included in this Table. As it can be noted, the results often show only modest activity against the parasites *in vitro* or against malaria in mice, suggesting that the species in question probably have only a limited effect in man and that cure of the disease is unlikely. However, this may not necessarily mean that medicines made from these species are of no value, since partially effective treatments might be beneficial in those cases that the course of the disease is shortened by reducing anaemia and lowering the risk of death or serious illness from other anaemia-related diseases. Moreover, benefits may also include the alleviation of symptoms such as pain and fever and immunomodulation leading to increased immunity [43]. Finally, it is important to stress that plant extracts could also be effective against the parasite on hepatic stage.

As commented before, the more recent development of artemisinin derivatives has re-affirmed the potential of plant species to provide effective drugs for the treatment of malaria. The antiplasmodial activity of all plant extracts depicted in Table 1 confirms this statement and suggests that it would be worthwhile to invest more time and resources into further investigation on active plant species identified in these studies.

**Table 1:** Recently-published plant extracts with antiplasmodial activity.

Family	Species	Extract (part)	Antiplasmodial Activity (IC_50_, μg/mL)		Strain	Ref.
Amaranthaceae	*Amaranthus spinosus*	Water (stem bark)	789.36 ± 7.19 ^¥^		*(N.S.)*^ ≠^ *^S^*	[44]
Annonaceae	*Uvariopsis congolana*	MeOH* (stem)	4.47 ± 0.45		W2*^ R^*	[45]
		MeOH* (leaves)	4.57 ± 0.76		W2*^ R^*	
	*Polyalthia oliveri*	MeOH* (stem bark)	4.30 ± 0.31		W2*^ R^*	
	*Enantia chlorantha*	MeOH* (stem)	4.79 ± 1.09		W2*^ R^*	
		MeOH* (stem bark)	2.06 ± 0.01		W2*^ R^*	
Aphloiaceae	*Aphloia theiforms*	MeOH (bark)	13.3 ± 0.8		3D7*^ S^*	[46]
			11.0 ± 3.1		W2*^ R^*	
		CH_2_Cl_2 _(bark)	16.1 ± 2.7		3D7*^ S^*	
			18.2 ± 2.7		W2*^ R^*	
		CH_2_Cl_2 _(leaves)	21.0 ± 1.6		3D7*^S^*	
			22.7 ± 2.9		W2*^ R^*	
Apiaceae	*Ferula oopoda*	MeOH (roots)	26.6		K1 *^R^*	[47]
			24.9		3D7*^ S^*	
	*Astrodaucus orientalis*	MeOH (aerial parts)	46.1		K1 *^R^*	
			42.6		3D7*^ S^*	
Apocynaceae	*Picralima nitida*	EtOH (seeds) [115 mg/Kg]	73.0%**		*(N.S.)* ^≠^ *^S^*	[48]
Asclepiadaceae	*Caralluma tuberculata*	Pet. Ether (aerial parts)	7.94	K1 *^R^*		[49]
Asteraceae	*Vernonia amygdalina*	EtOH (leaves)	9.7 ± 2.6	*(N.S.) ^S^*		[50]
		Pet. Ether (leaves)	2.5 ± 0.7	*(N.S.) ^S^*		
		Isoamyl alcohol (leaves)	2.7 ± 0.6	*(N.S.) ^S^*		
	*Psiadia arguta*	CH_2_Cl_2 _(leaves)	10.1 ± 2.2	3D7*^ S^*		[46]
			8.4 ± 1.1	W2*^ R^*		
		MeOH (leaves)	22.4 ± 2.2	3D7*^ S^*		
			26.1 ± 6.5	W2*^ R^*		
	*Centaurea bruguieriana*	MeOH (aerial parts)	36.9	3D7*^ S^*		[47]
	*Centaurea golestanica*	MeOH (aerial parts)	35.6	K1 *^R^*		
			31.6	3D7*^ S^*		
Boraginaceae	*Heliotropium zeylanicum*	MeOH (aerial parts)	8.41	K1 *^R^*		[49]
Buxaceae	*Buxus hyrcana*	MeOH (aerial parts)	4.7	K1 *^R^*		[47]
			7.7	3D7*^ S^*		
Caesalpiniaceae	*Cassia occidentalis*	EtOH (leaves)	2.8 ± 0.5	*(N.S.) ^S^*		[50]
		Pet. Ether (leaves)	1.5 ± 0.7	*(N.S.) ^S^*		
		Isoamyl alcohol (leaves)	18.6 ± 3.6	*(N.S.) ^S^*		
		CHCl_3_ [pH 2-3] (leaves)	2.9 ± 0.3	(N.S.) *^S^*		
Capparaceae	*Boscia angustifolia*	CH_2_Cl_2_ (leaves)	107.9	3D7*^ S^*		[51]
		MeOH (leaves)	37.6	3D7*^ S^*		
Caryophyllaceae	*Minuartia lineata*	MeOH (aerial parts)	44.0	3D7*^ S^*		[47]
Clusiaceae	*Croton zambesicus*	EtOH (roots) [81 mg/Kg]	86.18% **	ANKA ^≠^		[52]
		*n*-hexane (root) [57 mg/Kg]	57.88% **	ANKA ^≠^		
		CHCl_ 3_ (root) [57 mg/Kg]	75.39% **	ANKA ^≠^		
		AcOEt (root) [57 mg/Kg]	76.89% **	ANKA ^≠^		
		MeOH (root) [57 mg/Kg]	77.27% **	ANKA ^≠^		
	*Garcinia kola*	EtOH (stem bark)	2.9 ± 0.7	*(N.S.) ^S^*		[50]
		Pet. Ether (stem bark)	1.6 ± 0.2	*(N.S.) ^S^*		
		Isoamyl alc. (stem bark)	41.7 ± 3.2	*(N.S.) ^S^*		
		CHCl_3_ [pH 2-3] (stem bark)	27.1 ± 2.7	*(N.S.) ^S^*		
	*Symphonia globulifera*	MeOH (leaves)	4.1±0.5	K1 *^R^*		[53]
Combretaceae	*Terminalia bentzoe L.*	MeOH (fresh leaves)	12.8 ± 2.9	3D7*^ S^*		[46]
			12.8 ± 3.5	W2*^ R^*		
		CH_2_Cl_2 _(leaves)	42.7 ± 3.2	3D7*^ S^*		
			21.0 ± 2.1	W2*^ R^*		
Cucurbitaceae	*Momordica foetida*	Water (shoots)	40.7 ± 11.20	D10*^ S^*		[54]
			50.8 ± 3.00	K1 *^R^*		
		AcOEt (shoots)	30.0 ± 1.70	D10*^ S^*		
			29.30 ± 1.47	K1 *^R^*		
		MeOH (shoots)	75.4 ± 17.50	D10*^ S^*		
			68.80 ± 5.40	K1 *^R^*		
Dilleniaceae	*Tefracera pogge*	EtOH (leaves)	36.9± 4.2	*(N.S.) ^S^*		[50]
		Pet. Ether (leaves)	1.7 ± 0.4	*(N.S.) ^S^*		
		Isoamyl alcohol (leaves)	21.8 ± 5.2	*(N.S.) ^S^*		
Euphorbiaceae	*Euphorbia hirta*	EtOH (whole plant)	2.4 ± 0.2	*(N.S.) ^S^*		
		Pet. Ether (whole plant)	1.2 ± 0.3	*(N.S.) ^S^*		
		Isoamyl alc. (whole plant)	2.6 ± 1.2	*(N.S.) ^S^*		
	*Neoboutonia. glabracens*	MeOH (leaves)	5.50 ± 0.20	W2*^ R^*		[45]
	*Croton zambesicus*	MeOH (stem bark)	5.69 ± 0.06	W2*^ R^*		
	*Phyllantus niruri*	EtOH (whole plant)	2.5± 0.1	*(N.S.) ^S^*		[50]
		Pet. Ether (whole plant)	1.3 ± 0.3	*(N.S.) ^S^*		
		Isoamyl alc. (whole plant)	2.3 ± 0.5	*(N.S.) ^S^*		
Fabaceae	*Glycyrrhiza glabra*	MeOH (aerial parts)	17.5	K1 *^R^*		[47]
	*Erythrina fusca*	EtOAc (stem bark)	7.5	K1 *^R^*		[55]
	*Stylosanthes erecta*	CH_2_Cl_2 _(aerial parts)	21.9	3D7*^ S^*		[51]
		MeOH (aerial parts)	23.3	3D7*^ S^*		
	*Tetrapleura tetraptera*	EtOH (fruits) [900 mg/Kg]	76.37%**	*(N.S.)*^ ≠^ *^S^*		[56]
Geraniaceae	*Erodium oxyrrhnchum*	MeOH (aerial parts)	40.3	K1 *^R^*		[47]
			13.0	3D7*^ S^*		
Hypericaceae	*Harungana madagascariensis*	EtOH (stem bark)	0.052-0.517	*(N.S.)*		[57]
		MeOH (seeds)	3.6 ± 0.3	K1 *^R^*		[53]
Lamiaceae	*Otostegia persica*	MeOH (fruits + leaves)	31.1	K1 *^R^*		[47]
	*Otostegia michauxii*	MeOH (aerial parts)	44.6	K1 *^R^*		
	*Perovskia abrotanoides*	MeOH (aerial parts)	37.3	K1 *^R^*		
Loganiaceae	*Nuxia verticillata*	CH_2_Cl_2 _(leaves)	10.9 ± 1.8	3D7*^ S^*		[46]
			8.8 ± 1.2	W2*^ R^*		
		MeOH (leaves)	32.7 ± 7.4	3D7*^ S^*		
		CH_2_Cl_2 _(bark)	27.4 ± 6.6	3D7*^ S^*		
		MeOH (bark)	36.9 ± 5.7	3D7*^ S^*		
	*Buddleja salviifolia*	CH_2_Cl_2 _(bark)	49.9 ± 9.6	3D7*^ S^*		
		CH_2_Cl_2 _(leaves)	29.7 ± 12.6	3D7*^ S^*		
			18.6 ± 5.8	W2*^ R^*		
	*Strychnos angolensis*	EtOAc (roots)	17.0 ± 7.6	FCA20*^ S^*		[58]
	*Strychnos cocculoides*	EtOAc (leaves)	20.0 ± 11.9	FCA20*^ S^*		
	*Strychnos gossweileri*	EtOAc (roots)	12.4 ± 4.1	FCA20*^ S^*		
	*Strychnos henningsii*	EtOAc (leaves)	15.9 ± 3.0	FCA20*^ S^*		
	*Strychnos johnsonii*	EtOAc (stem)	16.4 ± 1.8	FCA20*^ S^*		
	*Strychnos mellodora*	EtOAc (leaves)	13.4	FCA20*^ S^*		
		MeOH (leaves)	29.5	FCA20*^ S^*		
		EtOAc (stem)	14.5 ± 1.5	FCA20*^ S^*		
		EtOAc (roots)	11.2 ± 3.6	FCA20*^ S^*		
		MeOH (roots)	25.4 ± 11.0	FCA20*^ S^*		
	*Strychnos scheffleri*	EtOAc (leaves)	21.2	FCA20*^ S^*		
	*Strychnos variabilis*	EtOAc (roots)	2.5 ± 0.2	FCA20*^ S^*		
		MeOH (roots)	2.3 ± 0.5	FCA20*^ S^*		
Meliaceae	*Trichilia emetica*	CH_2_Cl_2_ (leaves)	11.9	3D7*^ S^*		[51]
		MeOH (leaves)	47.6	3D7*^ S^*		
Mimosaceae	*Cylicodiscus gabunensis*	EtOH (stem bark) [60 mg/Kg]	83.3% ^¥^	*(N.S.)*^ ≠^ *^S^*		[59]
	*Albizia zygia*	MeOH (stem bark)	1.0 ± 0.1	K1 *^R^*		[53]
Moraceae	*Artocarpus communis*	MeOH (leaves)	4.00 ± 0.37	W2*^ R^*		[45]
Nyctagynaceae	*Boerhaavia erecta*	Water (stem bark)	564.95 ± 6.23 ^¥^	*(N.S.)*^ ≠^ *^S^*		[44]
Polygalaceae	*Securidaca longipedunculata*	CH_2_Cl_2_ (leaves)	6.9	3D7*^ S^*		[51]
Rubiaceae	*Vangueria infausta*	CHCl_3_ [fr.] (root bark)	3.8 ± 1.5	D6 *^ S^*		[60]
			4.5 ± 2.3	W2*^ R^*		
	*Morinda morindoides*	EtOH (leaves)	94.2 ± 3.4	*(N.S.) ^S^*		[50]
		Pet. Ether (leaves)	1.8 ± 0.2	*(N.S.) ^S^*		
		Isoamyl alcohol (leaves)	15.3 ± 3.6	*(N.S.) ^S^*		
		CHCl_3_ [pH 2-3] (leaves)	8.8 ± 2.5	*(N.S.) ^S^*		
Sapindaceae	*Cardiospermum halicacabum*	AcOEt (shoots)	28.60 ± 4.20	D10*^ S^*		[54]
			32.60 ± 2.60	K1 *^R^*		
		MeOH (shoots)	62.60 ± 9.40	D10*^ S^*		
			79.00 ± 5.20	K1 *^R^*		
Solanaceae	*Licium shawii*	MeOH (aerial parts)	7.75	K1 *^R^*		[49]
Tamaricaceae	*Tamarix aralensis*	MeOH (aerial parts)	43.8	3D7*^ S^*		[47]
Verbenaceae	*Lantana camara*	CH_2_Cl_2 _(leaves)	8.7 ± 1.0	3D7*^ S^*		[46]
			5.7 ± 1.6	W2*^ R^*		
Vitaceae	*Cissus quadrangulari*	CH_2_Cl_2 _(whole plant)	23.9	3D7*^ S^*		[51]
		MeOH (whole plant)	52.8	3D7*^ S^*		
Zingiberaceae	*Siphonochilus aethiopicus*	EtOAc (rhizomes)	2.90 ± 0.28	D10 *^S^*		[61]
			1.4	K1 *^R^*		

^¥ ^ED_50_ (mg/Kg); *^R^* Chloroquine-resistant strain; *^S^* Chloroquine-sensitive strain; *Acetogenine-rich methanol extract; **in vivo chemossupression; (N.S.) – Not specified; ^≠ ^*P. berghei berghei*, oral administration.

## 3. Antiplasmodial Non-Alkaloidal Natural Products

Although several classes of natural products are responsible for the antiplasmodial activity of many plant species used in traditional medicines for the treatment of malaria, the most important and diverse biopotency has been observed in alkaloids, quassinoids and sesquiterpene lactones [8]. Since alkaloids have been recently discussed in the literature [17,19], this review will highlight the majority of antiplasmodial non-alkaloidal natural products published in the period Jan/2008-May/2009 belonging to the classes of terpenes, limonoids, flavonoids, chromones, xanthones, anthraquinones, miscellaneous and related compounds.

### 3.1. Terpenes and related compounds

**Figure 3:**
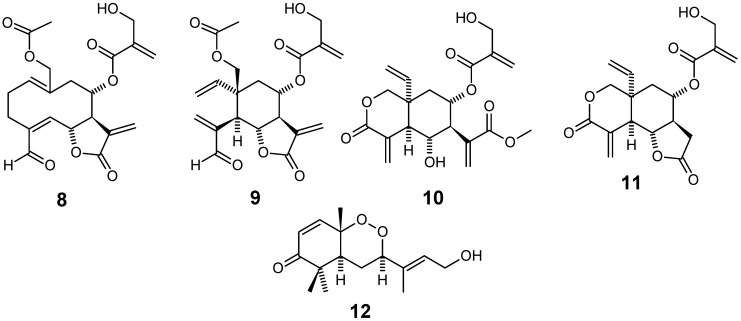
Antiplasmodial terpenes **8**-**12**.

Combined use of bioassay-guided fractionation based on *in vitro* antiplasmodial assay and dereplication based on HPLC–PDA–MS–SPE–NMR methods led to isolation of the sesquiterpene lactones **8**-**11** (Figure 3) as the main antiplasmodial constituents of the acetone extract of *Distephanus angulifolius* (Asteraceae), a scrambling shrub or climber commonly known as “Trailing Vernonia” due to its growth habit, which in South Africa is found from the Eastern Cape to Mozambique. The isolated compounds showed IC_50_ values in the range 1.6–3.8 μM and 2.1–4.9 μM against chloroquine sensitive D10 and chloroquine resistant W2 *P. falciparum* strains, respectively. Compounds **8** and **9** show slightly better effect than **10**, with **9 **having the lowest resistance index. Moreover, compounds **9** and **10** show a selectivity index 2.8 and 3.6 times higher, respectively, than **8**. In this work, resistance index has been defined as the ratio between the IC_50_ values of a given compound on W2/D10 strains, while selectivity index has been conceptualized as the ratio between IC_50_ values of such substance on D10 strain / Chinese Hamster Ovarian cell line. Under these concepts, a desirable chemotherapeutic lead structure should present both low resistance index and high selectivity index [62].

The CH_2_Cl_2_-MeOH extract of the roots of *Scleria striatinux* de Wild (syn. *S. striatonux*) (Cyperaceae), a local spice in parts of Cameroon, was shown to exhibit low activity against both D6 chloroquine-sensitive (IC_50_ = 80.4 µg/mL) and W2 resistant (IC_50_ = 89.4 µg/mL) strains of *P. falciparum.* A new and skeletally unique bicyclofarnesyl sesquiterpene endoperoxide, named okundoperoxide (**12**) (Figure 3), was isolated by bioassay-guided fractionation of extracts from this plant. This compound contains a cyclic endoperoxide structural moiety and was found to possess low antiplasmodial activity (IC_50_ around 176 - 180 µM for both strains) [63].

**Figure 4:**
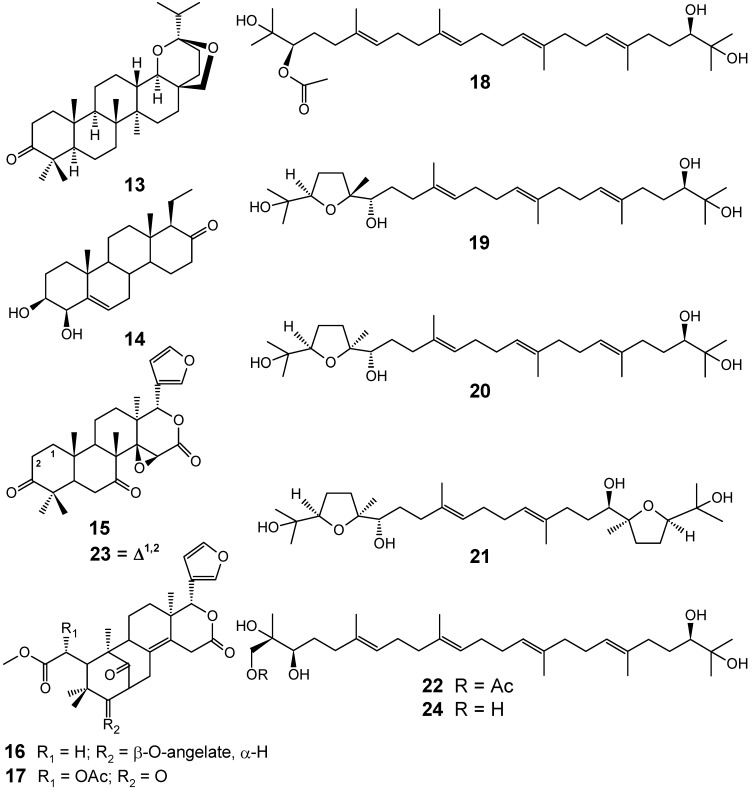
Antiplasmodial terpenes **13-24**.

From the stem bark of *Ekebergia capensis*, ten new triterpenoid compounds named ekeberins A (**13**), B (**14**), C1 (**15**), C2 (**16**), C3 (**17**), D1 (**18**), D2 (**19**), D3 (**20**), D4 (**21**) and D5 (**22**), were isolated together with the known compounds **23 **and **24 **(Figure 4), among 15 others. Several of these compounds were screened *in vitro* against both chloroquine-sensitive (FCR-3) and -resistant (K-1) *P. falciparum* isolates and were found to exhibit good antiplasmodial activity, with compounds **23 **(7-deacetoxy-7-oxogedunin) and **24 **(2-hydroxymethyl-2,3,22,23-tetrahydroxy-2,6,10,15,19,23-hexamethyl-6,10,14,18-tetracosatetraene) showing IC_50_ values of 6 and 7 μM, respectively. Compound **24 **at a dose of 500 mg/kg showed moderate parasitemia suppression of 52.9% against *P. berghei* NK 65 in a mouse model [64].

**Figure 5:**
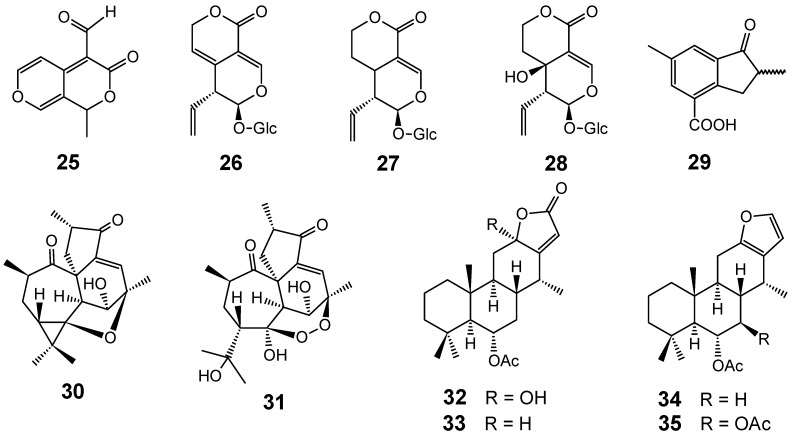
Antiplasmodial terpenes and related compounds **25-35**.

A secoiridoid aglycone with an atypical skeleton, named fagraldehyde (**25**), as well as the known secoiridoids gentiopicroside (**26**), sweroside (**27**) and swertiamarin (**28**) (Figure 5), were isolated from the bark and leaves of *Fagraea fragrans* (Gentianaceae) collected in Cambodia. Compound **25 **was weakly active *in vitro* against *P. falciparum*, exhibiting an IC_50_ value of 116.6 ± 9.4 μM (W2 strain), whereas compounds **26**-**28**, displaying IC_50_ values higher than 200 μM, were considered to be inactive [65].

A new indanone derivative **29** and two new diterpenoids **30** and **31 **(Figure 5), together with three known flavonoids, have been isolated from an ethanol extract of the leaves of *Croton steenkampianus* Gerstner (Euphorbiaceae), commonly known as “Marsh Fever-berry” and “Tonga Croton” in central and southern Africa. The isolated compounds were tested for their antiplasmodial activity and cytotoxicity. Antiplasmodial assays against chloroquine-susceptible strains (D10 and D6) and the chloroquine-resistant strains (Dd2 and W2) of *P. falciparum* showed that compound **30** gave good activities at 9.1-15.8 μM [66].

Japanese researchers have isolated two new cassane-type diterpenes, sucutiniranes A (**32**) and B (**33**), along with 6α-acetoxyvouacapane (**34**) and 6α,7β-diacetoxyvouacapane (**35**) (Figure 5) from the seeds of *Bowdichia nitida* (Fabaceae), a Brazilian Amazon species commonly known as “sucupira”. Compound **35** showed promising *in vitro* antiplasmodial activity against parasite *P. falciparum* 3D7 (IC_50_ = 1 µM) and a good selectivity index with regard to the cytotoxicity on COLO201 cells (IC_50_ > 250 µM), whereas other compounds were not active at a concentration of 3 µM (chloroquine: IC_50_ = 0.019 µM) [67].

**Figure 6:**
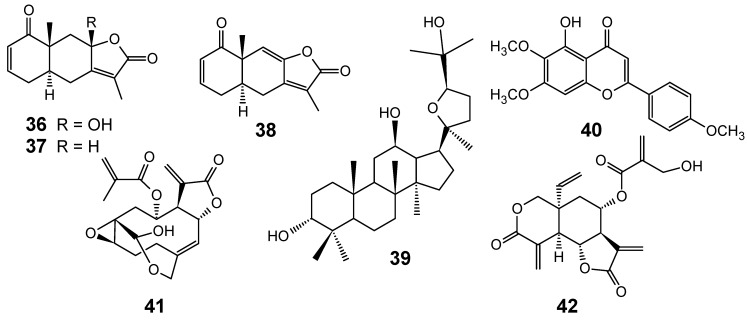
Antiplasmodial terpenes and related compounds **36-42**.

From the crude ethyl acetate extract of the medicinal plant *Siphonochilus aethiopicus* (Zingiberaceae), which showed a very good *in vitro* activity against the chloroquine-sensitive (D10) and chloroquine-resistant (K1) strains of *P. falciparum* with IC_50_ values of 2.9 and 1.4 µg/mL, respectively, a bioassay-guided fractionation led to the isolation of three novel furanoterpenoids **36**-**38** (Figure 6) with moderate/good *in vitro* antiplasmodial activity, displaying IC_50_ values of 73.0 µM (D10) and 67.3 µM (K1), for compound **36**; 109.4 µM (D10) and 25.2 µM (K1), for compound **37**; and 14.5 µM (D10) and 7.5 µM (K1), for compound **38**. The crude ethyl acetate extract has also showed a very good *in vivo* activity, with a parasitemia reduction comparable to chloroquine. The compounds and crude extract were more active against the K1 strain than the D10 strain of *P. falciparum* [61].

As *Salvia radula* (Lamiaceae) exhibited the best antiplasmodial activity (IC_50_ = 3.91 µg/mL) among 17 *Salvia* species used in traditional medicine in South Africa, it was selected for the bioassay-guided isolation of the active compounds. Fractionation of a 1:1 methanol/chloroform extract of this species afforded betulafolientriol oxide (**39**) and salvigenin (**40**) (Figure 6), which displayed similar or lower antiplasmodial activity [IC_50_ values = 4.95 and 24.60 μg/mL (10.4 and 74.9 μM), respectively] when compared to the crude solvent extract. The loss of activity may be due to synergy that might account for the better activity of mixture than isolated compounds [68].

From bioassay-guided fractionation of the crude acetone leaf extract of *Vernonia colorata* (Asteraceae), Chukwujekwu and co-workers isolated two sesquiterpene lactones (Figure 6) which displayed antiplasmodial activity against the D10 chloroquine-sensitive strain of *P. falciparum*: vernolide (**41**, IC_50_ = 5.16 μM) and vernodalin (**42**, IC_50_ = 1.44 μM), with selectivity indices of 1.02 and 2.79, respectively. In conclusion, the authors considered that these compounds were not shown to be specific antiplasmodial agents towards this parasite [69].

**Figure 7:**
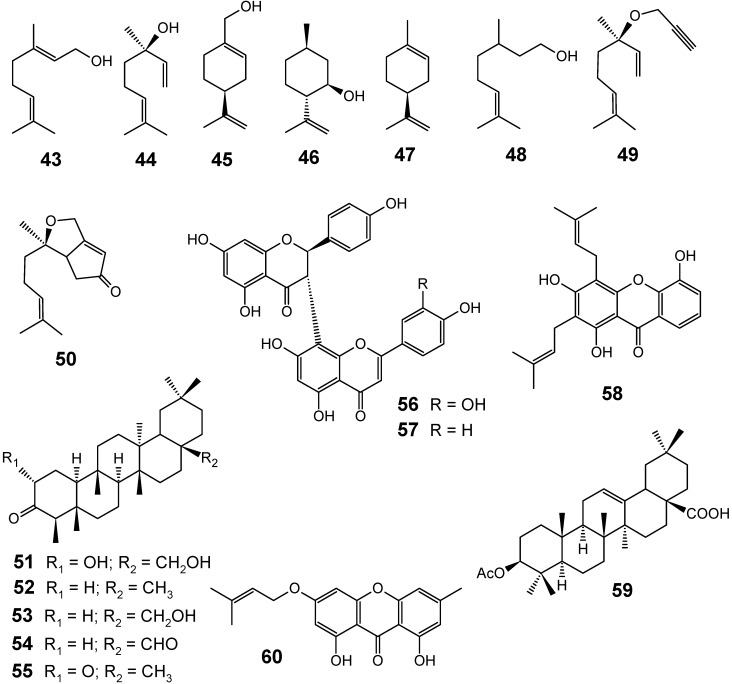
Antiplasmodial terpenes and related compounds **43-60**.

Pure natural monoterpenes geraniol (**43**), (-)-linalool (**44**), (-)-perillyl alcohol (**45**), (-)-isopulegol (**46**), (-)-limonene (**47**) and (±)-citronellol (**48**) (Figure 7) were evaluated *in vitro* for their antiplasmodial activities against the chloroquine-resistant FcM29-Cameroon strain of *P. falciparum*. Chemically modified terpenes were also prepared and assayed against the same strain in order to see whether the introduction of an alkyne, a cyclopropane, a diene, or a cyclopentenone moiety had an influence on their biological activity. The results showed the moderate-low activity of most natural monoterpenes (IC_50_ values ranging from 52 to 519 μM), geraniol (**43**, IC_50 _= 52 µM) and limonene (**47**, IC_50 _= 66 µM) being the most active ones in this study. However, the alkyne **49 **(IC_50 _= 39 µM) and cyclopentenone **50** (IC_50 _= 1.8 µM) derivatives showed a promising enhancement of activity compared with their parent molecules, reaching a 13- and 290-fold increase in the activity for **49** and **50**, respectively. Given the observed antiplasmodial activity of some of these modified monoterpenes, it was suggested that new monoterpene derivatives could be the basis for the research of new antimalarial drugs [70]. 

Although neither medicinal uses nor phytochemical studies had been reported before for *Endodesmia calophylloides* (Guttiferae), a tree found in Nigeria, Cameroon, Gabon and Angola, *n*-hexane (IC_50_ = 9.3 μg/mL), AcOEt (IC_50_ = 7.4 μg/mL) and MeOH (IC_50_ = 12.8 μg/mL) extracts from the stem bark of this plant showed potent antiplasmodial activities. A novel friedelane triterpenoid named endodesmiadiol (**51**), as well as the known compounds friedelin (**52**), canophyllol (**53**), canophyllal (**54**), cerin (**55**), morelloflavone (**56)**, volkensiflavone (**57**), 8-deoxygartanin (**58**), 3β-acetoxyoleanolic acid (**59**) and 1,8-dihydroxy-3-isoprenyloxy-6-methylxanthone (**60**) (Figure 7), were isolated from the ethyl acetate extract and assayed against the W2 chloroquine-resistant strain of *P. falciparum*. All compounds were found to be active with IC_50_ values ranging from 7.2 to 23.6 µM. From these results, the authors could anticipate arguing that friedelane derivatives might be interesting sources for new potential antimalarial leads [71].

**Figure 8:**
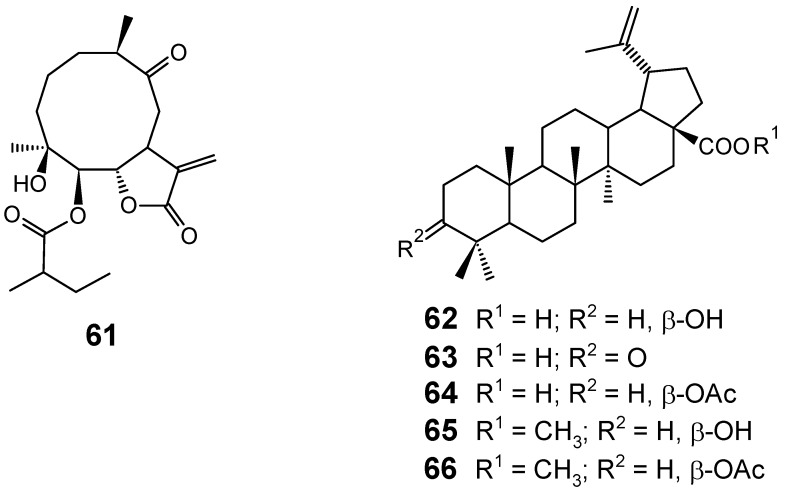
Antiplasmodial terpenes **61-66**.

The chloroform-soluble fraction obtained from the methanol extract of *Carpesium rosulatum* (Asteraceae) was found to have high *in vitro* antiplasmodial activity (IC_50_ = 8.2 μg/mL) against the chloroquine-resistant D10 strain of* P. falciparum*, this activity being attributed to Ineupatorolide A (**61**) (Figure 8), which displayed a very impressive antiplasmodial activity (IC_50_ = 0.019 µM) [72]. More recently, Korean researchers demonstrated that **61** shows potent *in vivo* antimalarial activity when tested against *P. berghei* in mice at doses of 2, 5 and 10 mg.kg^-1^·day^-1^, exhibiting a significant blood schizontocidal activity in 4-day early infection, repository evaluation and in established infection with a significant mean survival time comparable to that of the standard drug, chloroquine (5 mg·kg^-1^·day^-1^). According to the authors, compound **61** possesses a promising antiplasmodial activity which can be exploited in malaria therapy [73].

Betulinic acid (**62**) and its derivative compounds betulonic acid (**63**), betulinic acid acetate (**64**), betulinic acid methyl ester (**65**) and betulinic acid methyl ester acetate (**66**) (Figure 8) have been evaluated for their antimalarial properties. These substances showed antiplasmodial activity against W2 chloroquine-resistant *P. falciparum* parasites *in vitro*, with IC_50_ values of 9.89, 10.01, 5.99, 51.58 and 45.79 μM, respectively. Moreover, since betulinic acid acetate (**64**, SI = 9.6) displayed the best selectivity index among all substances tested, this compound was administered by intraperitoneal route to mice infected with *P. berghei*, causing a dose-dependent reduction of parasitemia of 70%, while mice treated with chloroquine had undetectable parasitemia. These results indicate that betulinic acid and its derivative compounds might be considered as potential lead compounds for the development of new antimalarial drugs, since the authors suggest that structural changes in the molecule significantly alter the anti-*Plasmodium* activity [74].

**Figure 9:**
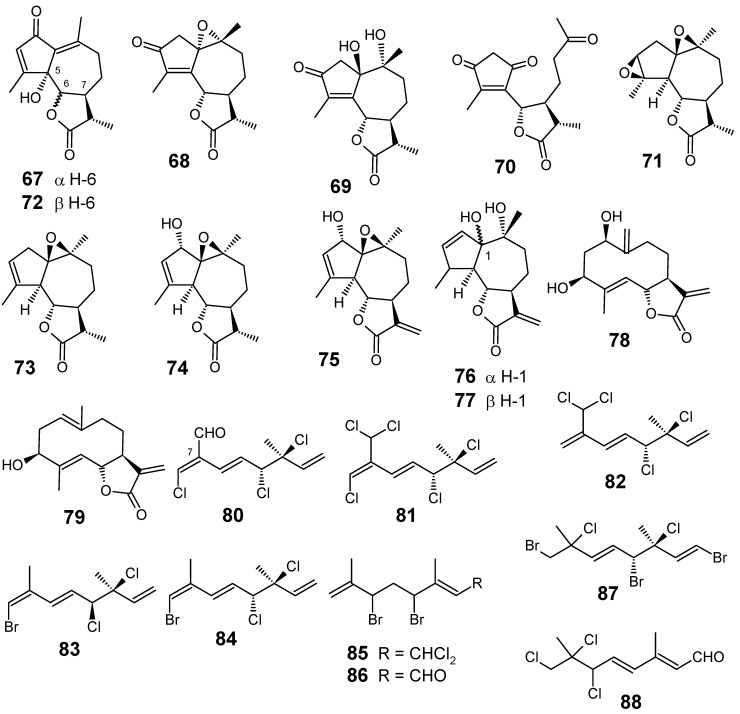
Antiplasmodial terpenes and related compounds **67-88**.

Leaves and flowers of *Artemisia gorgonum* (Asteraceae) collected in Fogo, Cape Verde islands, were phytochemically investigated, leading to the isolation and characterization of three new guaianolides **67**, **68**, **71**, and a secoguaianolide **70**, in addition to seven known guaianolides **69**, **72**-**77** and two known germacranolides **78** and **79 **(Figure 9). Most compounds exhibited mild antiplasmodial activities, ridentin (**79**) being the most interesting, with an IC_50_ of 15.3 ± 2.8 μM against *P. falciparum* FcB1 and weak cytotoxicity in a vero cell line (IC_50_ = 285.9 ± 15.7 μM) [75].

Three known **80**-**82** and two new halogenated monoterpenes **83** and **84**, all of them with a 3,7-dimethyl-3,4-dichloro-octa-1,5,7-triene skeleton, were isolated from the endemic marine red alga *Plocamium cornutum* (Turner) Harvey (Plocamiaceae) collected in South Africa. These compounds, together with compounds **85**-**88** (Figure 9) previously obtained from *P. corallorhiza*, were evaluated for their antiplasmodial activity against the chloroquine sensitive D10 strain of *P. falciparum*. Although the compounds tested were significantly less active than the standard compound (chloroquine, IC_50_ = 0.036 µM), it was interesting to note that compounds **81** and **82** containing the 7-dichloromethyl moiety were the most active ones (IC_50_ values of 16 and 17 µM, respectively), followed by compound **80** which contains an aldehyde functional group at this position. In addition, almost all the other compounds were essentially inactive, including plocoralide A (**85**) which contains a 1-dichloromethyl moiety. The importance of a functional group at position 7 is further emphasized when considering the lack of antiplasmodial activity of other related halogenated monoterpenes [76].

**Figure 10:**
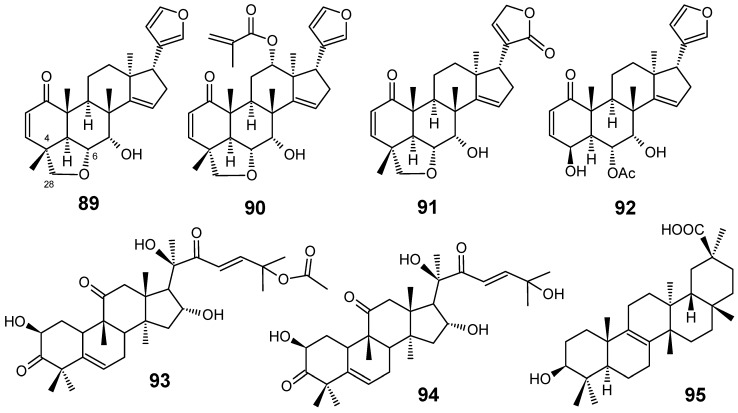
Antiplasmodial terpenes **89-95**.

Limonoids are highly oxidized secondary metabolites from Meliaceae species which are obtained through a unique biosynthetic route involving a tetranortriterpenoid nucleus. Numerous biological effects such as insecticidal, insect antifeedant, antibacterial, antifungal, antimalarial, anticancer and antiviral activities have been reported for them [77,78]. From the crude ethanol extract from the bark of *Chisocheton ceramicus*, Mohamad and co-workers isolated three new limonoids, named ceramicines B–D (**89**–**91**), together with ceramicine A (**92**) (Figure 10). Ceramicine B (**89**) showed a potent *in vitro* antiplasmodial activity against *P. falciparum* 3D7 (IC_50_ = 0.56 µM), whereas ceramicines C (**90**) and D (**91**) exhibited a relative good activity (IC_50_ = 4.83 µM and 5.06 µM, respectively) and ceramicine A (**92**) displayed a weak activity (IC_50_ = 100.37 µM). The higher antiplasmodial activity of compounds **89**-**91** than that of **92 **seems to be attributed to the presence of a tetrahydrofuran ring at C-4/C-6 and C-28 [79].

Starting from the first pharmacological screening of *Cogniauxia podolaena* (Cucurbitaceae) which confirmed that the roots of this species possesses antiplasmodial activity with IC_50_ values lower than 27 µg/mL for all extracts obtained with different solvents, a phytochemical study of this plant was undertaken and three triterpenes have been isolated: cucurbitacin B (**93**), cucurbitacin D (**94**) and 20-epibryonolic acid (**95**) (Figure 10). All isolated compounds were shown to have good antiplasmodial activity against the chloroquine-resistant FcM29 strain of *P. falciparum*, displaying IC_50_ values of 2.9, 7.7 and 4.3 µM, respectively. These results corroborate the conclusion that compounds **93**-**95** are the constituents responsible for the antiplasmodial activity of *C. podolaena* roots. In addition, compound **95 **showed a better selectivity index than those from **93** and **94**, indicating that this compound may be of therapeutic interest for further pharmacomodulation studies [80].

### 3.2. Flavonoids, chromones, xanthones, anthraquinones and related compounds

**Figure 11:**
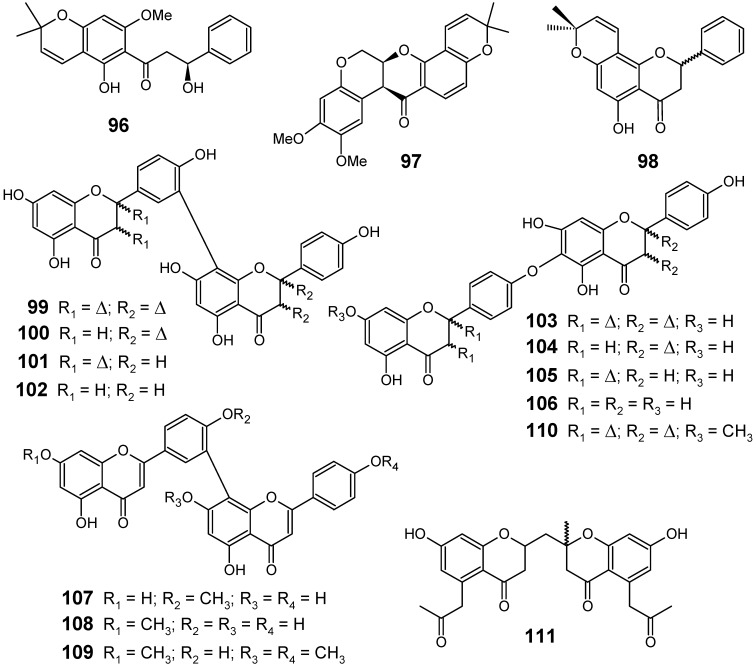
Antiplasmodial flavonoids and related compounds **96-111**.

From the seedpods of *Tephrosia elata* (Fabaceae), a new β-hydroxydihydrochalcone (**96**) was isolated, along with the known flavonoids deguelin (**97**) and obovatin (**98**) (Figure 11). The crude MeOH-CH_2_Cl_2_ (1:1) extract showed antiplasmodial activities against chloroquine-sensitive Sierra Leone I (D6) (IC_50_ = 8.4 ± 0.3 µg/mL) and chloroquine-resistant Indochina I (W2) (IC_50_ = 8.6 ± 1.0 µg/mL) strains of *P. falciparum*. 

Among the compounds isolated from the seedpods of *T. elata*, the new compound (**96**) exhibited the highest antiplasmodial activity with IC_50_ values of 8.2 ± 0.8 and 16.3 ± 0.9 µM against D6 and W2 strains, respectively, while the other substances (**97** and **98**) showed IC_50_ values ranging from 12.4 to 27.6 µM against these strains. This compound along with the other flavonoids appears to be responsible for the activities observed in the crude extract [81].

From a series of twelve biflavonoids **99**-**110** (Figure 11) containing amentoflavone and hinokiflavone derivatives isolated from the Indian medicinal herb *Selaginella bryopteris* (Selaginellaceae), eleven of them (**99-104**, **106-110**) were investigated for their antiprotozoal activity using *in vitro* assays against the K1 strain of *P. falciparum*, besides *Leishmania donovani*, *Trypanosoma brucei rhodesiense* and *Trypanosoma cruzi*. The highest antiplasmodial activity was displayed by **107** and **109** which exhibited an IC_50_ of 0.30 and 0.26 µM, respectively. To evaluate the *in vivo* activity against *P. berghei* of the most active compound (**109**), trimethylated amentoflavones were obtained by partial synthesis starting from amentoflavone (**99**). The synthesized mixture of trimethylated amentoflavones did not show activity in the *P. berghei* mouse model against female NMRI mice at 50 mg/kg by intraperitoneal administration [82].

A new bischromone, chrobisiamone A (**111**) (Figure 11), with a good *in vitro* antiplasmodial activity against parasite *P. falciparum* 3D7 (IC_50_ = 5.6 µM), has been isolated from the leaves of *Cassia siamea*, a Fabaceae species widely used in traditional medicine, particularly for the treatment of periodic fever and malaria in Indonesia [83].

In their studies on the screening of medicinal plants produced in Thailand with antimalarial activity against the multi-drug-resistant *P. falciparum* (K1 strain), Khaomek and collaborators have reported that an ethyl acetate (EtOAc) extract of the stem bark of *Erythrina fusca* Lour. showed significant antiplasmodial activity (IC_50_ = 7.5 µg/mL). 

**Figure 12:**
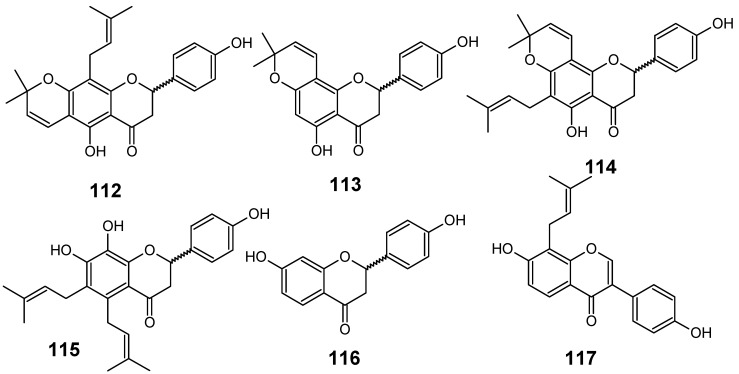
Antiplasmodial flavonoids **112-117**.

The flavonoids lupinifolin (**112**), citflavanone (**113**), erythrisenegalone (**114**), lonchocarpol A (**115**), liquiritigenin (**116**) and 8-prenyldaidzein (**117**) (Figure 12) were isolated from this extract, and three among them (**113**, **115** and **117**) were found to show *in vitro* antiplasmodial activity at a concentration less than 12.5 µg/mL. Diprenylated flavanone **115** (IC_50_ = 3.9 µM) displayed the most potent activity among the tested compounds, whereas **112** and **114** did not show such activity, even though these compounds also possess prenylated structures. This result postulates that appropriate introduction of prenyl groups into flavonoids may lead to more useful derivatives for construction of an antimalarial agent [55].

**Figure 13:**
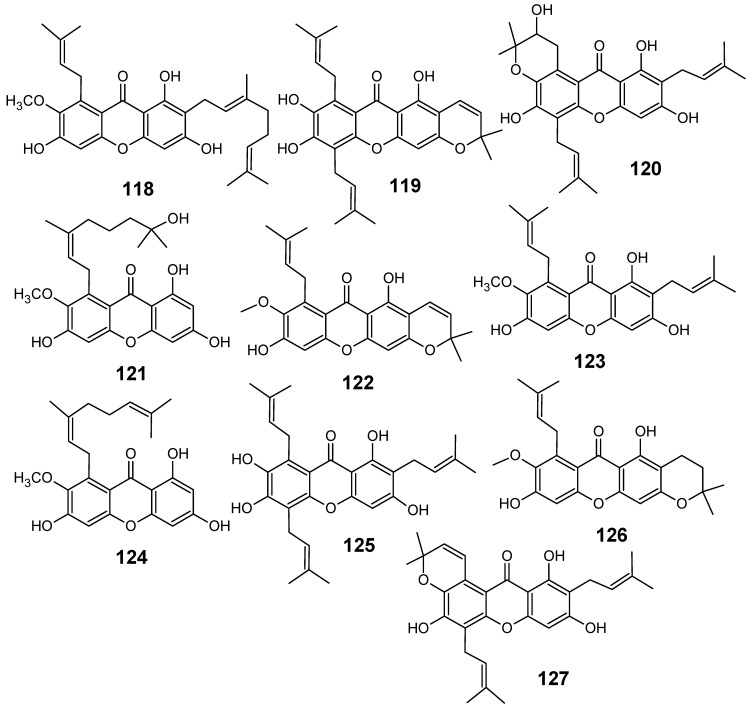
Antiplasmodial xanthones **118-127**.

From the stem bark of *Pentadesma butyracea* Sabine (Clusiaceae), a multipurpose rain forest species commonly called “butter tree” and widely distributed in tropical West Africa including Cameroon, four new xanthones named butyraxanthones A-D (**118**-**121**), together with six known xanthones (**122**-**127**) (Figure 13) and a triterpenoid (lupeol), were isolated and assayed *in vitro* for antiplasmodial activity against the *P. falciparum* chloroquine-resistant FcB1 strain. Except for compound **121 **(IC_50_ > 23.3 µM)**,** all of these xanthones exhibited good antiplasmodial activity with IC_50_ values ranging from 4.4 to 8.0 µM [84].

Chemical investigation of the methanol extract from the leaves of *Arrabidaea patellifera*, a Bignoniaceae plant from Panama, afforded mangiferin (**128**) and six new derivatives (**129-134**). 

**Figure 14:**
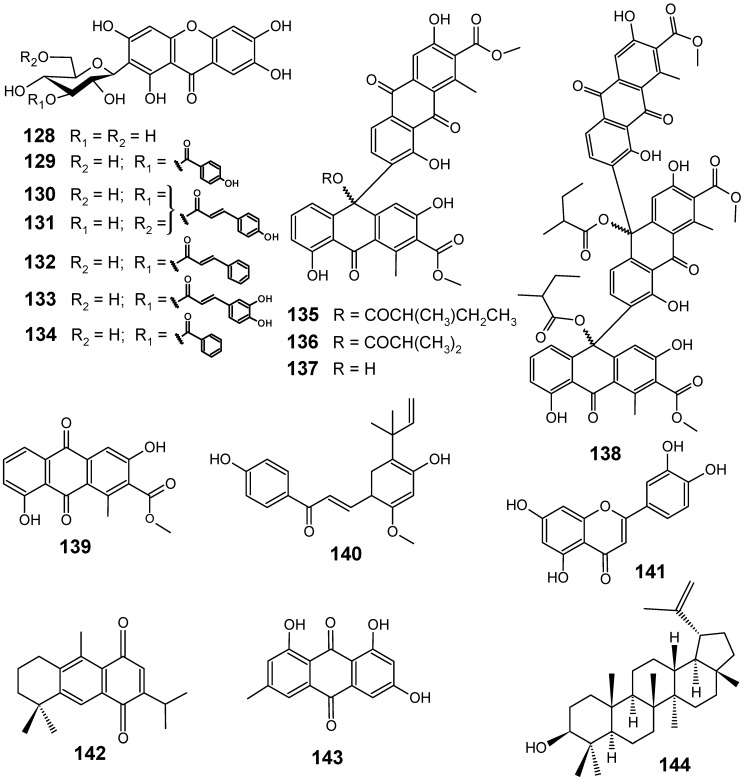
Antiplasmodial flavonoids and related compounds **128-144**.

Compounds **128-131** (Figure 14) were moderately active *in vitro* against chloroquine-sensitive *P. falciparum* 3D7 clone, displaying IC_50_ values of 23.8, 26.5, 18.1 and > 38.2 µM, respectively [85]. Bioassay-guided fractionation of the ethanol extract of a Madagascar collection of the bark of *Scutia myrtina* (Rhamnaceae) led to the isolation of three new anthrone–anthraquinones named scutianthraquinones A, B and C (**135–137**), one new bisanthrone–anthraquinone, scutianthraquinone D (**138**), and the known anthraquinone aloesaponarin I (**139**). Compounds **135-138** exhibited very good antiplasmodial activities against *P. falciparum* Dd2, with IC_50_ values ranging from 1.0 to 5.9 µM, while compounds **135** (IC_50 _= 1.7 µM), **136 **(IC_50 _= 7.9 µM) and **138** (IC_50 _= 5.0 µM) also exhibited significant antiplasmodial activities against *P. falciparum* FCM29 strain [86].

Interactions of two phytochemicals, artemisinin (**4**) and licochalcone A (**140**) (Figure 14), have been studied against synchronized erythrocytic stages of chloroquine-sensitive 3D7 and chloroquine-resistant RKL 303 strains of *P. falciparum*. These two compounds in combination showed synergistic antiplasmodial activity *in vitro* on these strains, provided by their antimalarial action on different metabolic pathways. According to the authors, artemisinin (**4**) but not licochalcone A (**140**) was shown to interfere with hemozoin formation, which could be either due to reduction in heme-detoxification or decrease in hemoglobin catabolism. The preferential inhibition of the *bc*1 complex of *P. falciparum* compared to that of mammals and the inhibition of new permeation pathways induced by the parasite in the host erythrocyte membrane have been suggested as the mode of action of licochalcone A (**140**) [87]. Neither of the phytochemicals alone or in combination obstructed sorbitol-induced hemolysis [88].

The effects of a range of common dietary flavonoids on the growth of two strains of *P. falciparum* have been investigated by Lehane and Saliba [89]. A chloroquine-sensitive (3D7) and a chloroquine-resistant (7G8) strain were tested for *in vitro* susceptibility to a range of individual dietary flavonoids and flavonoid combinations. Of the eleven flavonoids tested, eight showed antiplasmodial activity against the 3D7 strain (with IC_50_ values between 11 and 66 µM), and all showed activity against the 7G8 strain (with IC_50_ values between 12 and 76 µM). The most active compound against both strains was luteolin (**141**) (Figure 14), with IC_50_ values of 11 ± 1 µM and 12 ± 1 µM for 3D7 and 7G8, respectively. Luteolin was found to prevent the progression of parasite growth beyond the young trophozoite stage, and did not affect parasite susceptibility to the antimalarial drugs chloroquine or artemisinin. Combining low concentrations of flavonoids was found to produce an apparent additive antiplasmodial effect. The authors conclude that flavonoid combinations warrant further investigation as antiplasmodial agents [89].

The ethanol extract of *Zhumeria majdae* (Labiateae) showed good antiplasmodial activity *in vitro* against chloroquine sensitive (D6, Sierra Leone) and chloroquine resistant (W2, Indo China) strains of *P. falciparum*, with IC_50_ values of 8.8 and 7.5 μg/mL, respectively. Bioactivity-guided fractionation of this extract led to the isolation of 12,16-dideoxy aegyptinone B (**142**) (Figure 14), a rearranged abietane diterpene with a 1,4-naphthoquinone moiety, which exhibited a significant antiplasmodial activity with IC_50_ values of 4.4 and 4.7 µM against D6 and W2 strains, respectively. This compound was further found to have potent antileishmanial and mild cytotoxic activities [90].

*Cassia siamea* L. (Fabaceae), used traditionally in the southwest Nigerian ethnobotany as a laxative, for insomnia, diabetes and hypertension, has been recently identified as an antimalarial remedy [91,92]. This led to the bioassay-guided fractionation of the crude methanol stem bark extract from this plant, using the parasite lactate dehydrogenase assay and multi-resistant strain of *P. falciparum* (K1) for assessing the *in vitro* antimalarial activity. Emodin (**143**) and lupeol (**144**) (Figure 14) were isolated from the ethyl acetate fraction by a combination of chromatographic techniques. Both compounds were found to be the active principles responsible for the antiplasmodial property of *C. siamea*, with IC_50_ values of 18.5 µM for **143** and 11.7 µM for **144** [93].

**Figure 15:**
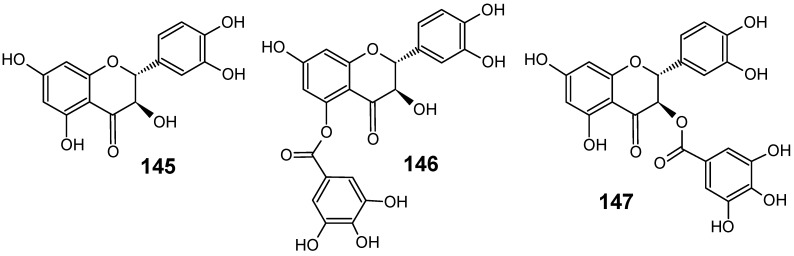
Antiplasmodial flavonoids **145-147**.

*Piptadenia pervillei* Vatke (Fabaceae) was selected from a screening programme devoted to the search of naturally-occuring antimalarial compounds from plants of Madagascar. Bioassay-guided fractionation of the ethyl acetate extract of the leaves led to the isolation of four phenolic compounds, (+)-catechin (**145**), (+)-catechin 5-gallate (**146**), (+)-catechin 3-gallate (**147**) (Figure 15) and ethyl gallate. Compounds **146 **and **147 **displayed the highest *in vitro* activity against the chloroquine-resistant strain FcB1 of *P. falciparum* with IC_50_ values of 1.2 μM and 1.0 μM, respectively, and no significant cytotoxicity against the human embryonic lung cells MRC-5 was measured (IC_50_ values > 75 μM). Ethyl gallate was less active with an IC_50_ value of 8.1 μM, and catechin **145 **showed no significant activity. Thus, the antiplasmodial activity of compounds **146**, **147 **and ethyl gallate appears to be associated with the presence of a gallate ester which plays an important role in inhibition against *P. falciparum* [94].

### 3.3. Miscellaneous compounds

**Figure 16:**
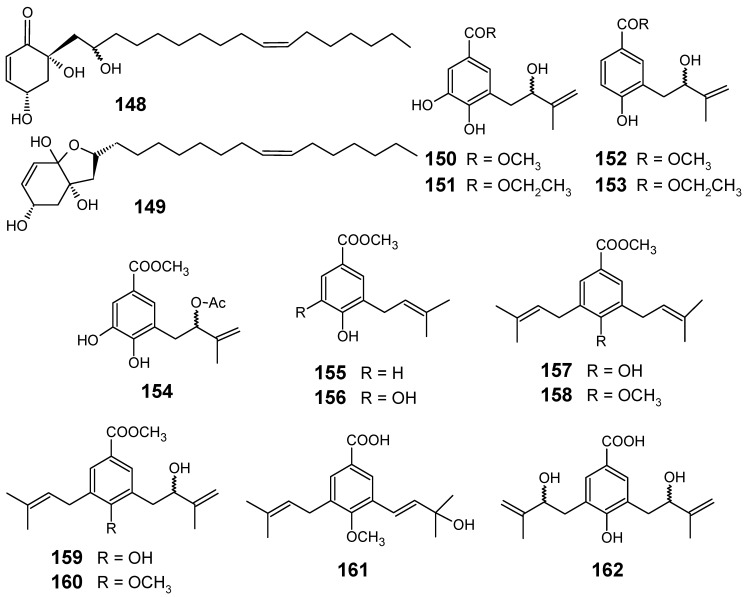
Antiplasmodial miscellaneous compounds **148-162**.

The bioassay-guided purification of CH_2_Cl_2_ bark extract of *Tapirira guianensis* (Anacardiaceae) led to the isolation of cyclic alkyl polyol derivatives **148** and **149 **(Figure 16), which were active against the chloroquine-sensitive F32 strain (IC_50_ = 4.7 µM) and the chloroquine-resistant FcB1 strain (IC_50_ = 5.4 µM) of *P. falciparum*. These results show that it is the **148-149** mixture which is mainly responsible for the activities of this plant extract, since other compounds isolated from *T. guianensis* did not show any interesting activity against the pathogens tested, although they were chemically quite similar [95].

*Piper glabratum* and *P. acutifolium* were analyzed for their content of main secondary constituents, affording nine new benzoic acid derivatives (**150**, **151**, **153**, **154**, **156**, and **159**-**162**), in addition to four known compounds (**152**, **155**, **157**, and **158**) (Figure 16). Compounds **150**, **152**, **155**, **156**, **157**, **159** and **161** were evaluated *in vitro* against *Leishmania* spp., *Trypanosoma cruzi* and *P. falciparum*. Among the evaluated compounds, only **155 **and **156 **exhibited a relative good antiplasmodial activity against F-32 Tanzania (chloroquine sensitive) strains of *P. falciparum*, with IC_50_ values of 12.7 and 16.3 μM, respectively. The rest of the compounds were considered less active (IC_50_ > 16.4 μM). Taking into account the results of the biological activities evaluated, a preliminary structure-activity relationship revealed that the prenylated 4-hydroxybenzoic acid derivatives with one side chain (**150**, **152**, **155** and **156**) were substantially more active as potential antiparasitic agents than those with two side chains (**157**, **158**, **159** and **160**) [96].

**Figure 17:**
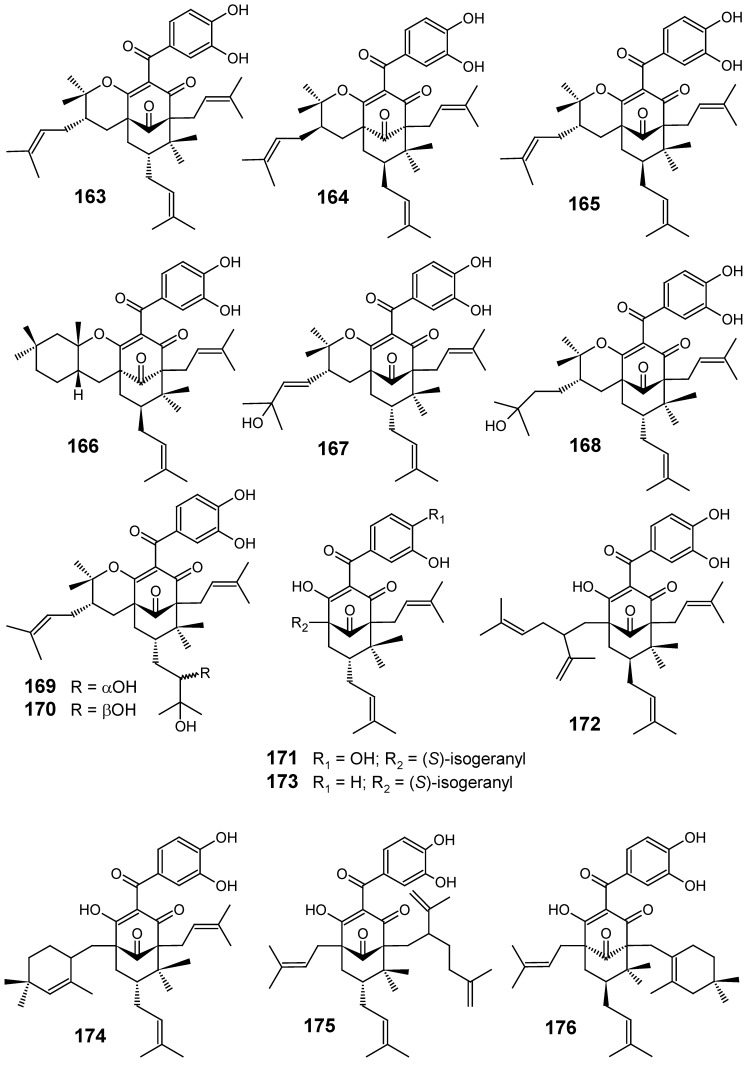
Antiplasmodial miscellaneous compounds **163-176**.

Eleven new polycyclic polyprenylated acylphloroglucinols (PPAPs) (**165**-**170**, **172**-**176**) along with the known isogarcinol (**163**), cycloxanthochymol (**164**) and garcinol (**171**) (Figure 17), were isolated from the methanol extract of the trunk latex of *Moronobea coccinea* (Clusiaceae) which showed 95% inhibitory growth at 10 μg/mL against a chloroquine-resistant strain of *P. falciparum* (FcB1). Compounds **163**-**176** exhibited good antiplasmodial activity, being compounds **163**-**170** the most active ones with their IC_50_ values ranging from 3.3 to 9.0 μM. These results indicated that the benzophenones with a tetrahydropyran ring are the most potent compounds. In contrast, compounds **171**-**176** showed lower antiplasmodial activities with IC_50_ values over 10 μM [97].

**Figure 18:**
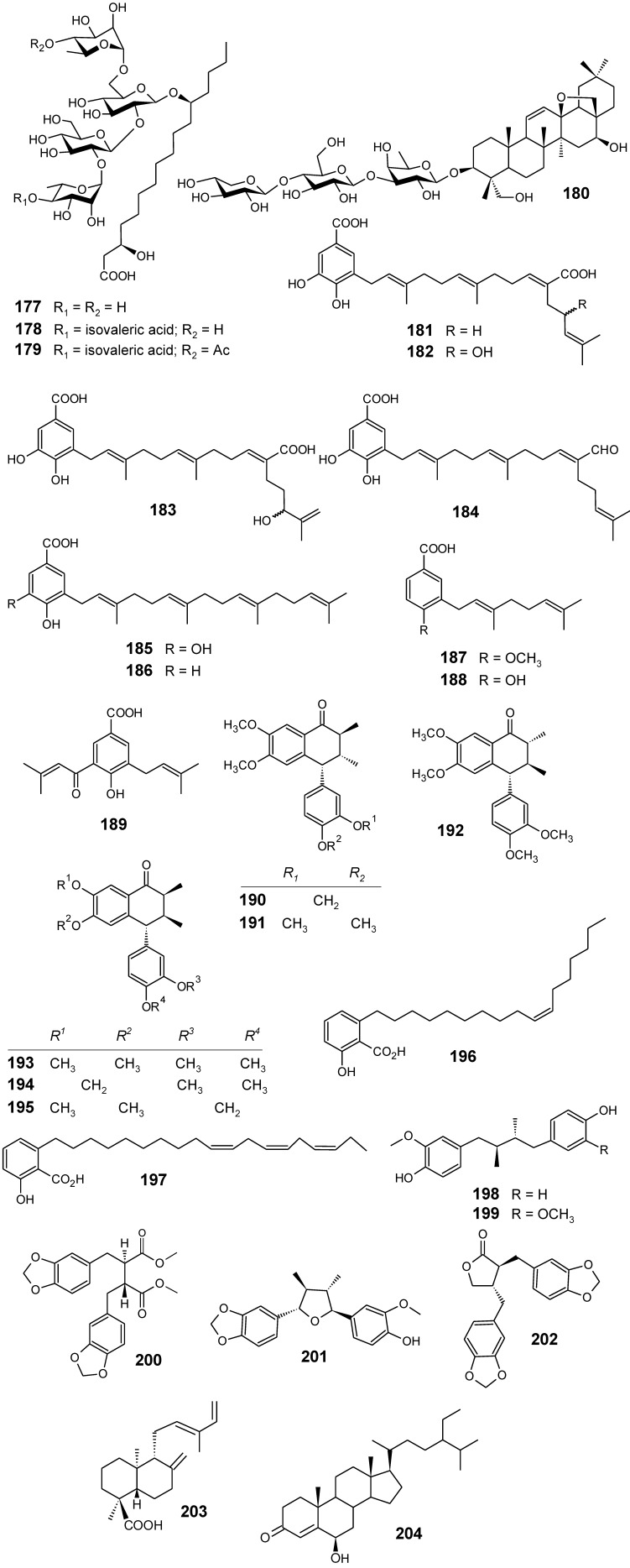
Antiplasmodial miscellaneous compounds **177-204**.

From compounds such as crypthophilic acids A–C (**177**-**179**) (Figure 18), which are the first resin glycosides occurring in another family (Scrophulariaceae) that is not Convolvulaceae, along with buddlejasaponin III (**180**), L-tryptophan and other constituents isolated from the aerial parts of *Scrophularia cryptophila*, only compounds **179**, L-tryptophan and **180** showed antimalarial activity against K1 strain of *P. falciparum* with IC_50_ values of 4.0, 81.3 and 24.5 μM, respectively. In target identification studies, none of the three compounds inhibited *Pf*FabI, a key enzyme from the *P. falciparum* type II fatty acid cascade, FAS-II. In addition, all compounds were found to be selective as they did not possess any cytotoxicity on mammalian L6 cells even at high concentrations (IC_50_ values >> 470 μM) [98].

Fractionation of dichloromethane extracts from the leaves of *Piper heterophyllum* and *P. aduncum* afforded three new prenylated hydroxybenzoic acids (**181**-**183**), along with six known compounds (**184**-**189**) (Figure 18). Evaluation of the antiparasitic activity for all isolates showed that all the compounds were considered to be moderately active (IC_50_ >> 10 µg/mL), except for compound **181**, which exhibited a good activity against *P. falciparum* with an IC_50_ of 7.0 µM, in comparison with chloroquine used as a positive control (IC_50_ = 0.3 µM). In conclusion, a geranylgeranyl side chain along with a carboxyl acid group as in **181** seem to be relevant for the antiplasmodial activity [99].

Extracts from *Holostylis reniformis* were tested *in vivo* against *P. berghei* and *in vitro* against a chloroquine-resistant strain of *P. falciparum*. The hexane extract of the roots was the most active, causing 67% reduction of parasitemia *in vivo*. From this extract, six lignans **190**-**195 **(Figure 18), including a new (7’*R*,8*S*,8’*S*)-3’,4’-methylenedioxy-4,5-dimethoxy-2,7’-cyclolignan-7-one (**195**), were isolated and assayed *in vitro* against *P. falciparum.* The three most active lignans (**190**-**192**) showed IC_50_ values of <0.32 µM (<0.12 µg/mL), lignan **192** being the most active compound (IC_50_ = 0.20 µM). Lignan **194** exhibited a lower activity (IC_50_ = 8.00 µM), whereas lignan **195** did not exhibit any activity under the same experimental conditions. An evaluation of minimum lethal dose (30%) values showed low toxicity for these lignans in a hepatic cell line (Hep G2A16). Therefore, *in vivo* assays and clinical studies need to be undertaken with these compounds in order to confirm them as potential candidates for the development of antimalarial drugs [100].

Based on previous work which revealed that petroleum ether extracts of *Viola websteri* (Violaceae) showed inhibition value of 31.7 as percentage of parasite inhibition at 25 μg/mL, this extract was investigated for its activity against the chloroquine-sensitive D10 strain of *P. falciparum*. Bioassay-guided fractionation led to the identification of two structurally related compounds (**196 **and **197**) (Figure 18) which were shown to be responsible for the observed activity of the extract. The main antiplasmodial principles, 6-(8’*Z*-pentadecenyl)-salicylic acid (**196**) and 6-(8’*Z*, 11’*Z*, 14’*Z*-heptadecatrienyl)-salicylic acid (**197**), have been isolated from *V. websteri* for the first time. In addition, this is the first report on the antiplasmodial activity of these compounds. Although compounds **196 **(IC_50_ = 10.1 ± 3.2 µM) and **197 **(IC_50_ = 13.3 ± 6.7 µM) are over 100 times less active than the positive controls, the data suggest that their antiplasmodial activity might not be due to general toxicity [101].

The dichloromethane, methanol and aqueous ethanol extracts of the stem bark of *Pycnanthus angolensis* (Myristicaceae) were evaluated for their *in vitro* activity against the 3D7 *P. falciparum* strain. The CH_2_Cl_2_ extract was the most active showing an IC_50_ = 1.6 μg/mL. From this extract, a new dibenzylbutane lignan, *threo*-4,4′-dihydroxy-3-methoxylignan (**198**) named pycnantolol, together with the known lignans (-)-dihydroguaiaretic acid (**199**), heliobuphthalmin (**200**), talaumidin (**201**), hinokinin (**202**), the labdane type diterpene ozic acid (**203**) and the steroids stigmast-4-en-6β-ol-3-one (**204**) (Figure 18), β-sitosterol and stigmasterol were isolated. The antiplasmodial activity of lignans **198–202**, ozic acid (**203**) and stigmast-4-en-6β-ol-3-one (**204**) was evaluated against 3D7 and Dd2 *P*. *falciparum* strains. In contrast with the crude extracts, these isolated compounds have not shown significant antiplasmodial activity against both strains, the lowest IC_50_ value (60.5 µM) being obtained for talaumidin (**201**) against the Dd2-chloroquine resistant *P*. *falciparum* strain. These results might be explained by synergistic effects between the different constituents of the complex extract and, especially if the antimalarial activity remains being observed in clinical studies and/or animal models, they could suggest that a standardization of the bark extract might be the best solution to a rational use of this traditional antimalarial plant [102].

## 4. Perspectives and Remarks on the Development of New Drugs and Phytomedicines for Malaria

The research on new antimalarial agents presently faces two distinct challenges: the search for new chemical entities (NCE) of natural or synthetic origin, and the development of phytomedicines [19]. The antiplasmodial/antimalarial natural products focused in this review can be divided into two groups: one of highly active compounds, of complex structures, for which no possibility of practical synthesis can be forseen, and another one, of moderate to low activity but of relative simple structures and, therefore, their synthesis and/or of their analogues could be undertaken. Plant species producing compounds of the first group are potential candidates for the development of phytomedicines, while the second group would represent templates to synthetic drugs.

Will the research on traditional plants contribute for the discovery of new antimalarial drugs? There is no doubt about this possibility. Atovaquone, quinine, artemisinin and its semi-synthetic derivatives are remarkable examples of the diverse contribution of natural products for the development of effective antimalarial drugs, particularly valuable for the treatment of chloroquine-resistant parasites. 

Indeed, several potent antiplasmodial natural products have been described, as shown in this review, most of them have only been evaluated by *in vitro* assays and few of them were evaluated for cytotoxicity and still smaller is the number of those assayed *in vivo*. Besides, many of them are found in low concentrations in the plant species and usually as part of complex mixtures making their isolation and purification highly expensive. These are typical situations which point to the development of phytomedicines, the known active compounds being useful as chemical-biological markers to guarantee product quality. 

Validation of traditional plant remedies presents limitations such as prioritization of plant species for research, lack of informations on ethnobotany of these plants (location and abundance, parts used, form of use, duration of treatment), definition of dosages due to variation on the concentrations of active ingredients in a plant species [103]. It should also be stressed that a basic requirement for the validation of a medicinal plant is the standardization of extracts to be evaluated, including the identification and quantification of chemical and/or biological markers to assure the development of efficient and safe phytomedicines in a short time and at low cost. It is well known that the qualitative and quantitative contents of secondary metabolites in a plant are susceptible to marked variation that is regulated by intrinsic factors (ontogeny and phenology) as well by abiotic (*e.g.* light, moisture, nutrient availability) and biotic factors such as different physiological and growth stages [104], which make the standardization obligatory.

## 5. Conclusions

This work reviewed most recently-published non-alkaloidal natural compounds from plants with antiplasmodial and antimalarial properties, besides the majority of antiplasmodial crude extracts published in the last five years. Considering the many antiplasmodial activities observed for all crude extracts and natural compounds described here, it can be stated that in fact there are optimistic perspectives on the continuing investigation of plants used in traditional medicines for the treatment of malaria, and they will certainly lead the scientific community to the discovery of more new efficient molecular templates and phytomedicines for this disease. Malaria and other neglected diseases, such as Chagas’ disease, leishmaniasis and African trypanosomiasis, among others, have a devastating impact on the world’s poor. Unfortunately they have been progressively marginalized by those in charge of making research programme decisions both in public and private sectors, since people suffering from these diseases have not offered a market lucrative enough to attract any notable investment in research and development for new drugs. Thus, this work also intends to stimulate and bring together new and intensive efforts from all research communities of the world to the quest of efficient phytomedicines and novel potential drug candidates both for malaria and other neglected diseases.
